# Integrating Taxonomic, Functional and Phylogenetic Beta Diversities: Interactive Effects with the Biome and Land Use across Taxa

**DOI:** 10.1371/journal.pone.0126854

**Published:** 2015-05-15

**Authors:** Julian Martin Corbelli, Gustavo Andres Zurita, Julieta Filloy, Juan Pablo Galvis, Natalia Isabel Vespa, Isabel Bellocq

**Affiliations:** 1 Departamento de Ecología, Genética y Evolución, IEGEBA, (CONICET-UBA), Facultad de Ciencias Exactas y Naturales, Universidad de, Buenos Aires, Ciudad Universitaria, Pabellón 2, Piso 4, CA Buenos Aires (1428), Argentina; 2 Instituto de Biología Subtropical—Facultad de Ciencias Forestales, Universidad Nacional de Misiones—CONICET, Bertoni 85, Pto Iguazú (3770), Misiones, Argentina; 3 División Paleozoología Invertebrados, Facultad de Ciencias Naturales y Museo (FCNyM), Universidad Nacional de La Plata, Museo de Ciencias Naturales de La Plata, Paseo del Bosque s/n. La Plata (1900), Buenos Aires, Argentina; Scientific Research Centre, Slovenian Academy of Sciences and Arts, SLOVENIA

## Abstract

The spatial distribution of species, functional traits and phylogenetic relationships at both the regional and local scales provide complementary approaches to study patterns of biodiversity and help to untangle the mechanisms driving community assembly. Few studies have simultaneously considered the taxonomic (TBD), functional (FBD) and phylogenetic (PBD) facets of beta diversity. Here we analyze the associations between TBD, FBD, and PBD with the biome (representing different regional species pools) and land use, and investigate whether TBD, FBD and PBD were correlated. In the study design we considered two widely used indicator taxa (birds and ants) from two contrasting biomes (subtropical forest and grassland) and land uses (tree plantations and cropfields) in the southern Neotropics. Non-metric multidimensional scaling showed that taxonomic, functional and phylogenetic distances were associated to biome and land use; study sites grouped into four groups on the bi-dimensional space (cropfields in forest and grassland, and tree plantations in forest and grassland), and that was consistent across beta diversity facets and taxa. Mantel and PERMANOVA tests showed that TBD, FBD and PBD were positively correlated for both bird and ant assemblages; in general, partial correlations were also significant. Some of the functional traits considered here were conserved along phylogeny. Our results will contribute to the development of sound land use planning and beta diversity conservation.

## Introduction

A major goal in ecological research is to explain patterns of biological diversity in natural and anthropogenic environments. Traditional approaches from the purely taxonomic viewpoint recognized three spatial components of diversity: local and regional species diversity (alpha and gamma, respectively) and species turnover (beta diversity). However, since the beginning of the XXI century studies started to focus on the spatial distribution of both species functional traits and phylogenetic relationships [1], and more recently on mechanisms beyond those patterns of biodiversity at different temporal and spatial scales [2].

Community ecologists increasingly recognize that a trait-based approach may be more meaningful than the species richness or composition to understand species responses to the environment [3]. The diversity of traits, or functional diversity, represents the diversity of species’ niches or functions [3,4], and has been used to understand how diversity respond to environmental disturbances [5,6] and how species diversity relates to ecosystem function [4–8]. Thus, traits determine where a species can live, how species interact, and the species contribution to ecosystem functioning [9]. Furthermore, the phylogenetic dimension of biodiversity reflects evolutionary differences among species based on times since divergence from a common ancestor [10]; it represents an estimate of phylogenetically conserved ecological and phenotypic differences among species [11]. The study of phylogenetic diversity provides insight into how evolutionary and ecological processes may interact to shape patterns of species and trait richness and composition [12]. Thus, the study of biodiversity is no longer limited to the taxonomic perspective, but it has been expanded to understand functional and phylogenetic changes within and between communities. Functional and phylogenetic diversities are related to ecosystem resilience to environmental disturbances [13], and conservation objectives are expanding to include multiple facets of diversity and ecosystem services [14]. The three facets of diversity may show different patterns of change along successional stages [15], and land use may affect functional structure of communities that is not necessarily reflected by the taxonomic diversity [16]. Here we integrate the taxonomic, functional and phylogenetic approaches to the study of beta diversity.

Beta diversity is a central concept in theoretical ecology, conservation biology, and ecosystem management [17]. While taxonomic beta diversity (TBD) was defined as the change in species composition across geographical space [18,19], functional beta diversity (FBD) was defined as the change in ecological functions or species traits between assemblages [20]; and phylogenetic beta diversity (PBD) as a measure of how deep lineages occurring in different assemblages have been separated in evolutionary time [21]. The simultaneous study of the three facets of beta diversity might reveal phylogenetically basal or terminal turnover between communities (for example, the turnover of phylogenetically close species would be considered low phylogenetic but high taxonomic turnover), and the phylogenetic signal in trait data (in such a case, the phylogenetic turnover between communities should mirror the functional turnover) [12]. Thus, an integrated approach of TBD, FBD and PBD can improve our understanding on how biodiversity patterns are caused and maintained, and the long term consequence of human disturbances on biological assemblages and ecosystem functioning [22]. For example, Devictor *et al*. [23] showed the congruence between patterns of the three beta diversity facets of birds suggesting the application for delimitation of regional ecotones. Flynn *et al*. [15] found that facets of plant beta diversity were correlated but only functional turnover showed significant deviations from random expectations along succession after human disturbance, suggesting successional changes in the process of assemblage formation and the relevance to consider all facets of diversity even though they may be correlated.

Anthropogenic pressure on terrestrial ecosystems has been accelerated in the last decades, associated to an increase on human incomes and population growth [24]. Human activities that require large extensions of land, such as agriculture and forestry, often result in habitat conversion due to land use for cropfields and tree plantations. In intensively modified landscapes where little natural habitat remains, human activities promote the replacement or impoverishment of native communities and the arrival of cosmopolitan species (loss of beta diversity); consequently, it is expected that taxonomic similarity between communities increases in this process of biotic homogenization [25]. Furthermore, given that species able to exploit human-modified habitats tend to be ecologically redundant and/or phylogenetically close related, FBD and PBD would also be lost [26]. How habitat replacement and land-use intensification change patterns of TBD, FBD and PBD remains little explored.

Climate acts as a regional filter that sorts species distribution according to each species range of tolerance to the various environmental factors, a process of assemblage formation known as species sorting at the metacommunity scale [27,28]; together with the species dispersal and interspecific competition determines the species presence-absence at a given site. That complex process results on different regional biomes and species pools over which human activities impose additional filters. Different types of human land uses impose different local filters to each pool of species and their functional traits. Here we analyze the associations between taxonomic, functional and phylogenetic turnover with biomes (representing different regional species pools) and human land uses (representing different environmental filters), identify the species contributing the most to differences in assemblage composition, and investigate whether TBD, FBD and PBD were positively correlated. In the study design we considered two widely used indicator taxa (birds and ants) from two contrasting biomes (subtropical forest and grassland) and human land uses (tree plantations and cropfields). Our working hypothesis is that species ecological niche facing environmental filtering (a deterministic process) is the primary driver of assemblage formation at both the regional and local levels. Thus, if the species capability of responding to regional and local environmental conditions were phylogenetically conserved and were different for each biome and land use, for both ants and birds we predict that 1) taxonomic, phylogenetic and functional differentiation between communities is determined by a combination of the effects of the regional context and local habitat and 2) TBD, FBD and PBD are positively correlated.

## Materials and Methods

### Study design

To study the turnover between biological assemblages, we tested the multivariate response of community composition to biome and land use, based on the taxonomic, functional and phylogenetic similarities between sites located in different regions and human land uses in the southern Neotropics. We selected two conservation priority biomes (subtropical forest and temperate grassland) with contrasting climates and vegetation structure, and two extended land uses (soybean cropfields and mature eucalypt plantations) with contrasting vegetation structure. In each biome, we selected five study sites per land use, for a total of 10 sites per biome. Birds and ants were used as independent biological models to consider community responses of different organisms. Field study did not involve endangered or protected species, and no birds were collected.

### Study areas and sites

**Fig 1:**
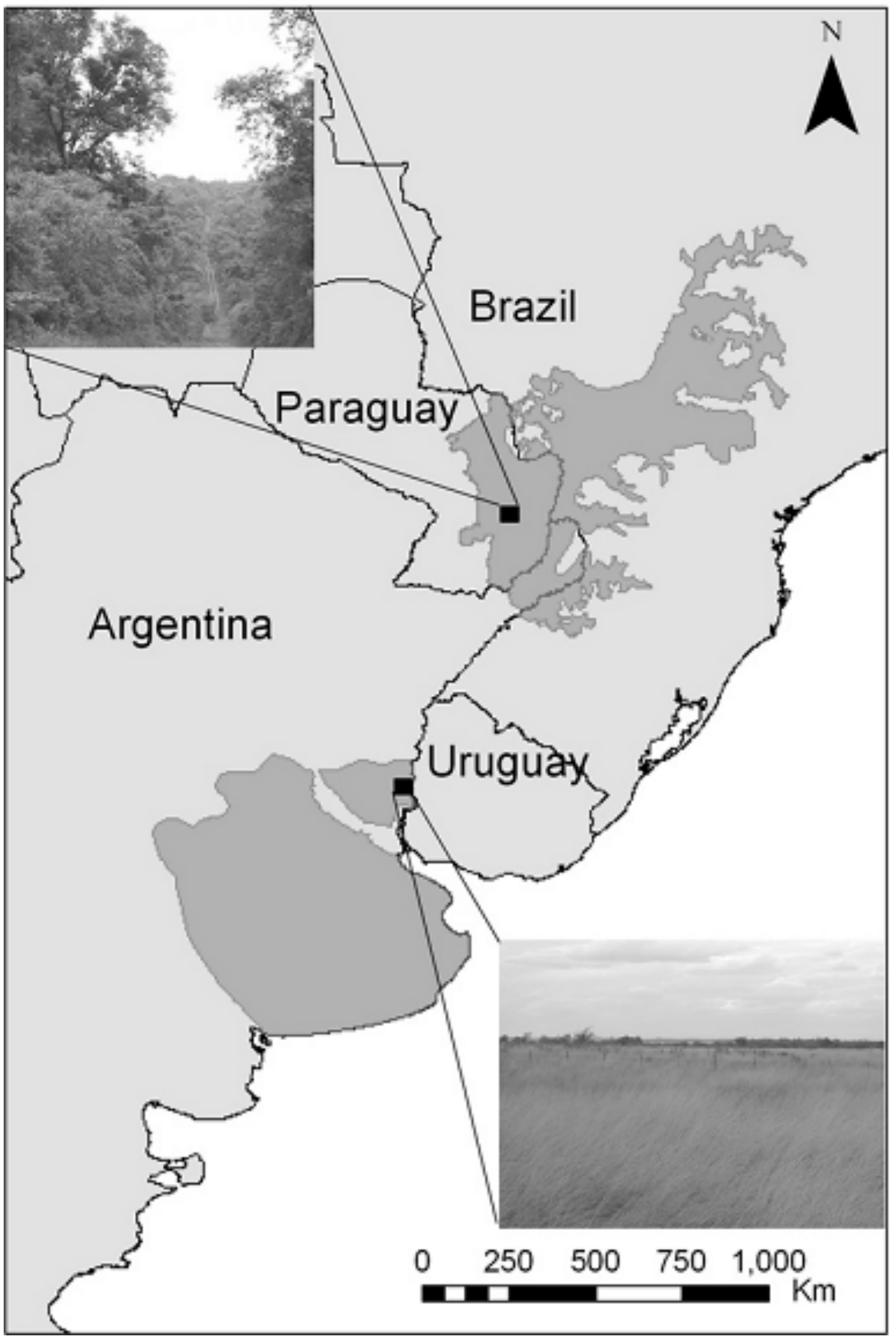
Physiognomy and geographical location of study biomes (Atlantic Forest in the North and Pampean grassland in the South) and study sites in the southern Neotropics.

The two selected biomes were the semideciduous subtropical Atlantic forest and the Pampean grassland (from here on Forest and Grassland, respectively) in southern South America (Fig 1); they are both considered priority biomes for biodiversity conservation [29,30]. Study sites were located in the eastern Paraguay area of the Atlantic Forest [31], and in the Mesopotamic Pampa in eastern Argentina [32,33] (Fig 1). The Atlantic forest in Paraguay was originally occupied by semideciduous forests; the climate is subtropical with average annual temperatures of 20°C and average annual rainfall of 2000 mm [34]. In the eastern Paraguay area, only 13% of the original native forest remains [35], and it concentrates 80% of the soybean cropfields of the country [36]. Both cropfields and tree plantations replaced the more or less degraded native forests over the last 50 years. The Pampean region was originally a grassland crossed by ravines; the climate is temperate, average annual temperature is 15°C, and annual rainfalls range from 1000 mm in the North to 600 mm in the South [31]. The Pampas have a history of antropogenic use; it was first used for extensive ranching followed by agriculture increasing in intensity over the 20th century. Currently, native vegetation is highly degraded and fragmented [37]; tree plantations replaced cattle pastures and cropfields in some areas.

In both Forest and Grassland, we selected five soybean cropfields and five stands of mature eucalypt plantations (from here on cropfields and tree plantations). Within each biome, sampling sites were located at an average distance of 16 km in Grassland and 10 km in Forest. Sampling sites with the same land use (soybean and eucalypt) within each region were located at an average distance of 11 km in Grassland and 9 km in Forest (S1 Table). When sampling started, soybean cropfields were two months-old, and eucalypt plantations were 7–8 years old. Soybean usually rotates with corn. Agricultural and silvicultural management (e.g. herbicide application, thinning) was similar between biomes. The study was carried out on private lands, and the heads of the following forest companies or landowners should be contacted for future permissions: Desarrollos Madereros S.A., Tierra Roja S.A., Estancia El Palmar, Paul Forestal S.R.L., Aserradero Ubajay de Siete Hnos. S.R.L., La Aurora del Palmar, Mastellone Hnos. S.A., Redepa S.A.

### Bird surveys and ant sampling

Bird surveys and ant collections were conducted in December 2007 in Forest and during January 2008 in Grassland, determined by soybean phenology. Birds were surveyed by establishing 10 observation points (200-m apart to avoid sub-sample overlap) in the 20 study sites. At each observation point, we recorded all birds seen or heard within a 100 m radius and five minutes observation period, on a single visit during the breeding season [38]. Surveys were simultaneously conducted by two trained independent observers from dawn to 10:30 on clear and sunny mornings. We verified that six to eight observation points in each study site were enough to detect 75–100% of the species recorded by a sampling effort of 10 observation points and five minutes time-period [39].

Ants were sampled during 28 consecutive days in each study site, by using 10 pitfall traps [40] located 10-m apart along a transect. Each trap consisted of a plastic container (500 ml volume, 85 mm diameter) with 150 ml of a propylene glycol and: water (1:2) solution. Species and morphospecies were identified following Bolton [41] nomenclature and taxonomic keys (S2 Table).

### Selection of functional traits

We selected functional traits related to the recorded species life-history, based on literature studying functional diversity or responses to habitat replacement by birds [42– 45] and ants [46–48]. We selected nine traits for birds adapted from Lopez-Lanus *et al*. [49] and Stotz *et al*. [50] and four traits for ants based on Andersen [51] and Fernández [52] (Table 1).

**Table 1:** Bird and ant functional traits considered in the estimation of functional beta diversity.

Birds	**AHS**, amplitude of habitat use (one to five habitats; more than five habitats); **TNA**, trophic niche amplitude (generalist; specialist); **RP**, reproductive potential (one to three eggs; more than three eggs); **SHD**, sensitivity to human disturbance (unfavored; favored); **BD**, body size (less than 100 gr, more than 100 gr); **AHT**, association with habitat type (grasslands: **GRA**; forests: **FOR**);**DIE**, diet (**FRU**: frugivore-granivore; **INS**, insectivore; **CAR**, carnivorous bird of prey; **OMN**, omnivore); **FS**, foraging stratum (**HIGH**; **LOW**); **MIG**, migratory status (resident; migratory)
Ants	**FG**, functional group (**C**, cryptic; **T**, specialist in tropical climate; **W**, specialist in warm climate; **SP**, specialist predators; **SC**, subordinate camponitines; **GM**, generalist mirmicines; **O**, opportunist; **DD**, dominant dolicoderines); **DIE**, diet (**SP**, specialist predator; **FV**, fresh vegetation; **GF**,generalist forager; **E**,exudate collector; **GP**, generalist predator; **GRA**, granivore); **HAB**, association with habitat type (**GRA**, grasslands; **FOR**, forests); **SIZ**, worker body size (**S**, small; **M**, medium; **L**, large)

### Construction of phylogenetic super-trees

We constructed two informal super-trees including all recorded species of birds and ants. Informal super-trees combine different phylogenies by taxonomic substitution, i.e. terminal taxa in one tree are replaced by trees representing phylogenetic relationships within each taxon [53]. Bird super-tree topology was obtained from Hackett *et al*. [54]. Then, the recorded species were added by taxonomic substitution following Birdsley, Irestedt *et al*., Fjdelsa *et al*., Ericson *et al*., Irestedt *et al*., Jonsson and Fjdelsa, Lerner and Mindell, Brown, Tree of Life Web Project, Harshman, Mindell and Harshman, Baker and Pereira, Brown and Mindell, and Moore and Miglia [55–70].

Ant super-tree was first assembled combining phylogenies by Moreau *et al*. and Brady *et al*. [71,72]. Then, taxonomic substitutions were done using phylogenies given by Schultz and Brady, Brandão and Mayhe-Nunes and Wild [73–75]. Species absent from reference phylogenies were assembled within related taxa, based on bird [50] and ant [41] systematics (S1 Fig).

### Data analysis

We performed a series of analysis to 1) statistically (PERMANOVA, PERMDISP) and visually (NMDS) explore the independent influence of biome and land use on patterns of taxonomic, functional and phylogenetic beta diversities (2x2 factorial design); 2) explore the individual ant and bird species contributing mostly to differentiate land uses within and between biomes (SIMPER); and 3) examine the associations among the different facets of diversity (simple and partial Mantel tests) and the existence of phylogenetic signal in trait data (D-Statistic).

The three beta diversity facets were estimated using the appropriate distance measure between sites. To estimate TBD between land uses (cropfields and tree plantations) and biomes (Forest and Grassland) we first built an incidence matrix (sites x species) for each of birds and ants, in which species presence/absence was recorded for each study site; then we calculated the 1-Sorensen index as a measure of taxonomic dissimilarity. To estimate FBD and PBD for both birds and ants, we first built phenotypic dendrograms and phylogenetic ultrametric trees and then calculated the 1-Sorf and 1-PhyloSor indices using PICANTE [76]. Sorf and PhyloSor represent the proportion of branch lengths shared by two assemblages [77]; they are analogous to the Sorensen taxonomic similarity index, and consequently we minimized the potential lack of correlation between TBD, FBD and PBD due to differences in index construction.

The phenotypic distance (euclidean) matrix (species x species), used to calculate the 1-Sorf index, was built using the selected species functional traits as variables (Table 1). All traits were defined as binary categorical variables, and multi-state traits (such as diet, habitat type, worker ant body-size, or ant functional group) were analyzed as multiple binary characters (0 = no, 1 = yes) [44]. Using phenotypic distances between species, we performed a hierarchical clustering procedure (UPGMA) in R [78] to obtain the functional dendrogram. To calculate 1-Phylosor, phylogenetic ultrametric trees and phylogenetic distance matrices (species x species) were obtained after branch length adjustment in each constructed super-tree. Adjustments were conducted using *bladj* algorithm (Phylocom, [79]), which minimizes the variance between branch lengths within the constraints imposed by the dating of tree internal nodes. Node ages were obtained from available information for birds [80] and ants [71–73] compiled on the Time Tree of Life website [81].

After estimating TBD, FBD and PBD we tested for significant associations between taxonomic, functional and phylogenetic distance matrices with biome and land use. To do that, we first visually explored site ordination based on taxonomic, functional and phylogenetic distances between assemblages in the Euclidean space, by performing non-metric multidimensional scaling (NMDS). First and second axes were plotted to evaluate whether site ordination was associated with biome or land use. Then, we performed permutational multivariate analysis of variance (PERMANOVA) [82] that can be computed for any distance index and allows to test for interaction effects between factors (i.e. biome x land use). However, the interaction term may have significant effects on distances when simple effects are different in direction or magnitude; thus, we tested for significant simple effects and for homogeneity of multivariate dispersion using PERMDISP [83]. Significance was obtained for each test by 9999 Monte Carlo permutations. Finally, the Similarity Percentage analysis (SIMPER) [84] was performed to identify species that contributed mostly to the taxonomic 1-Sor distances between treatments. SIMPER performs pairwise comparisons of groups of sampling units (treatments; i.e. cropfields in Grassland) and ranks all species according to the average contribution of each one to the overall average distance index. Using presence data, species that occur in most sites within treatments are those that contribute the most to the similarity within and dissimilarity between treatments. Thus, SIMPER allows identifying bird and ant species that better discriminate between treatments [85]. NMDS and SIMPER were implemented in R [78].

To analyze the association between the three beta diversity facets, we performed correlations between pairs of distances matrices (i.e., simple Mantel tests) [86] to test the association between each pair of taxonomic (1-Sor), functional (1-Sorf) and phylogenetic (1-PhyloSor) beta diversities. Then, we performed partial correlations (i.e., partial Mantel tests) between pairs of distance matrices [87] to remove the effects of the third distance matrix. Tests were performed using the VEGAN package [88] applicable in R [78], and 1000 permutations of the distance matrices to obtain the significance level.

A high correlation between 1-Sorf and 1-Phylosor is a strong indicator of phylogenetic signal in trait data [89]. Thus, the phylogenetic conservation of species traits was explored by testing the degree of phylogenetic signal of each bird and ant species trait using D-statistic for binary traits [90]. Starting from trait values randomly distributed along a phylogenetic tree (D ~ 1), the D-statistic approaches zero as trait phylogenetic signal increases. When traits are more conserved than expected by the Brownian evolutionary model (i.e. trait values differ proportionally to species divergence times [91]), then the D-statistic is significantly less than zero. Observed and expected distributions of the D-statistic, and significance level for each test were obtained using the CAPER package [92] applicable in R [78].

## Results

We recorded a total of 638 individual birds representing 49 species (S3 Table). For the 49 recorded species, we built a functional dendrogram with 47 internal nodes and assembled a phylogenetic tree with 41 internal nodes (S1 Fig). For ants, we captured over 25,000 individuals from 35 genera, 84 species and 15 morphospecies (S3 Table). Among the total 99 ant species and morphospecies, 28 had no references on the preferred habitat type (forests or grasslands); thus, they were excluded when testing the association of functional distances between biome and land use, and the correlation between functional and taxonomic or phylogenetic distances. Consequently, we built a functional dendrogram for 71 species or morphospecies with 51 nodes and a phylogenetic tree for all 99 species and morphospecies with 61 internal nodes (S1 Fig).

**Fig 2:**
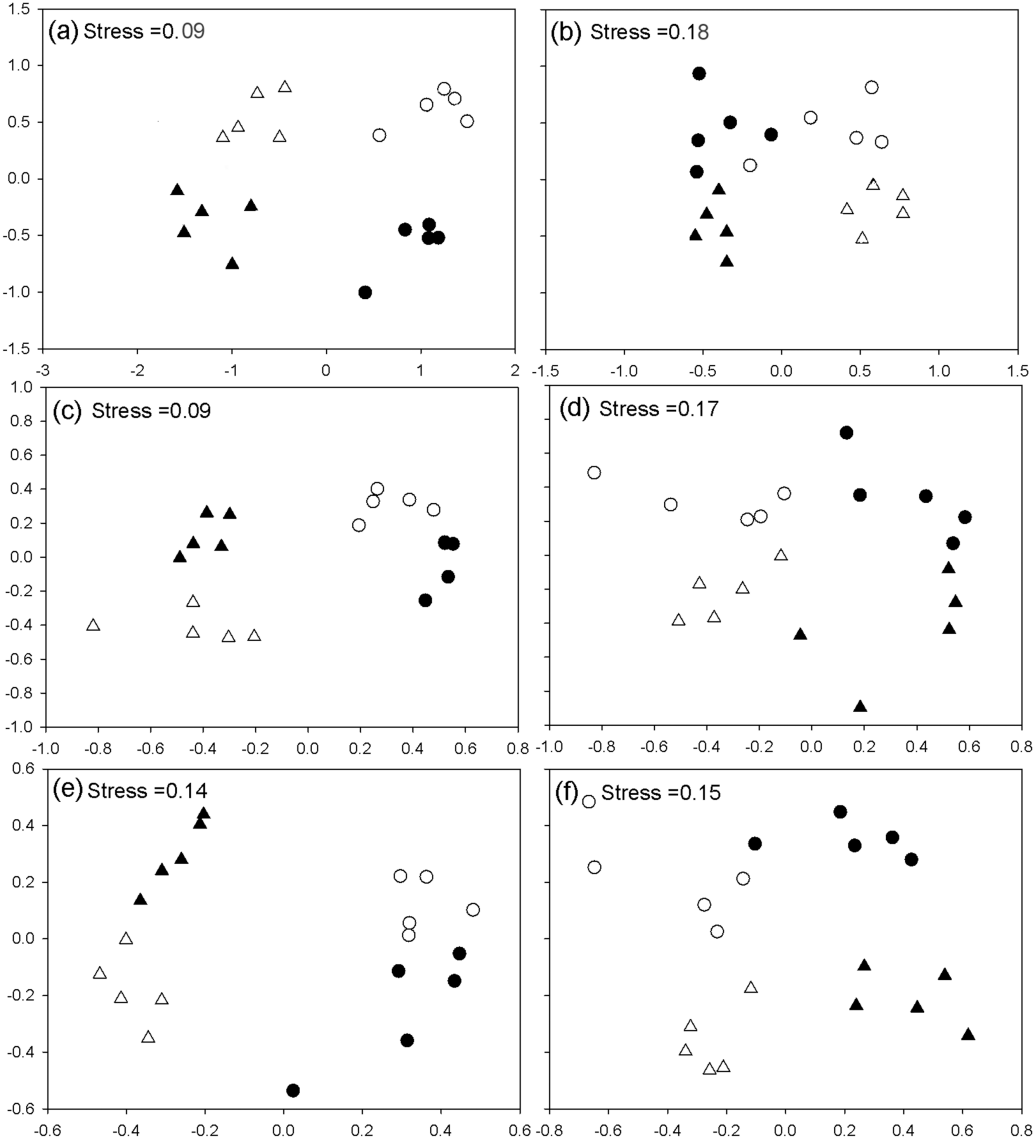
Non-metric multidimensional scaling (axes NMDS1 vs. NMDS2) using the taxonomic 1-Sorensen (a and b), functional 1-Sorf (c and d) and phylogenetic 1-PhyloSor (e and f) distances between bird (left) and ant (right) assemblages from soybean cropfields (circles) and mature eucalipt plantations (triangles) located in the Atlantic Forest (filled symbols) or the Pampean grasslands (empty symbols).

Taxonomic, functional and phylogenetic distances between bird and ant assemblages were associated to biome and land-use in the NMDS (Fig 2). Study sites grouped into four clearly differentiated groups on the bi-dimensional space (cropfields in Forest and Grassland, and tree plantations in Forest and Grassland). Site grouping was consistent across taxa and beta diversity facets indicating similar patterns of changes in the taxonomic composition, functional traits, and phylogenetic lineages.

For both bird and ant assemblages, PERMANOVA and single effects tests confirmed that site taxonomic, functional and phylogenetic composition was associated to both land use and biome (Table 2). PERMANOVA detected significant interactions between biome and land use for the three distance indices, and single effect tests showed significant effects of biome on each level of land use and viceversa. PERMDISP applied to bird dataset showed that multivariate dispersion among eucalypt plantations was lower in Forest than in Grasslands for taxonomic 1-Sor distance (t = 2.8, p = 0.02), functional 1-Sorf distance (t = 2.7, p = 0.02), and phylogenetic 1-PhyloSor distance (t = 2.5, p = 0.03). For ants, PERMDISP showed no differences in multivariate dispersion between treatments for the taxonomic 1-Sor (F = 0.7, p = 0.47). Furthermore, PERMDISP showed that multivariate dispersion among tree plantations was lower in Forest than in Grasslands for the phylogenetic 1-PhyloSor (t = 4.2, p<0.01) and the 1-Sorf distances (t = 2.6, p = 0.02). SIMPER analyses revealed the species of birds and ants that made important contributions to the taxonomic distance between treatment levels (Table 3).

**Table 2:** PERMANOVA tests (p-values) for birds and ants and the taxonomic (1-Sor), functional (1-Sorf) and phylogenetic (1-PhyloSor) distance indices.

			Biome single effects t (p)		Land-use single effects t (p)	
	Distance index	Interaction term (biome x land use). F(p)	In soybean cropfields	In eucalypt plantations	In Atlantic Forest	In Pampean Grassland
Birds	1-Sor	8.9 (<0.01)	3.0 (<0.01)	2.8 (<0.01)	5.2 (<0.01)	3.9 (<0.01)
	1-Sorf	9.1 (<0.01)	2.8 (<0.01)	2.9 (<0.01)	5.1 (<0.01)	3.9 (<0.01)
	1-PhyloSor	8.5 (<0.01)	2.4 (<0.01)	3.3 (<0.01)	3.8 (<0.01)	3.9 (<0.01)
Ants	1-Sor	3.7 (<0.01)	5.1(<0.01)	3.9 (<0.01)	2.1 (<0.01)	2.2 (<0.01)
	1-Sorf	0.8 (0.5)	9.9 (<0.01)		7.1 (<0.01)	
	1-PhyloSor	2.9 (0.01)	2.3 (<0.01)	2.6 (<0.01)	2.0 (<0.01)	2.5 (<0.01)

**Table 3:** Proportion of occurrence of bird and ant species that contributed the most to the distance between treatment levels (land use x biome combinations).

		Prop. occur				Prop. occur		
Pair of treatments	Bird species	A	B	Contribution (%)	Ant species	A	B	Contribution (%)
Soybean Forest(A) vs. Soybean Grassland(B)	*Volantinia jacarina*	1	0	6.23	*Strumigenys lousianae*	1	0	4.17
	*Zonotrichia capensis*	0	1	6.23	*Ectatomma bruneum*	0	1	4.17
	*Crypturellus parvirostris*	0.8	0	4.89	*Mycetarotes parallelus*	0	0.8	3.27
					*Pyramica eggersi*	0	0.8	3.23
					*Labidus praedator*	0	0.8	3.23
					*Mycocepurus goeldii*	0	0.8	3.23
					*Ectatomma edentatum*	0.2	0.8	3
					*Pachycondyla striata*	0.8	0.2	2.96
					*Pogonomyrmex naegelli*	0.8	0.2	2.79
Soybean Forest(A) vs. Eucalypt Forest(B)	*Ammodramus humeralis*	1	0	4.86	*Ectatomma bruneum*	1	0	4.55
	*Buteo magnirostris*	0	1	4.86	*Pogonomyrmex coartactus*	0	0.8	3.76
	*Guira guira*	0	1	4.86	*Paratrechina silvestrii*	0.8	0	3.61
	*Pitangus sulphuratus*	0	1	4.86	*Mycetarotes parallelus*	0.8	0	3.56
	*Troglodytes aedon*	0	1	4.86	*Pyramica eggersi*	0.8	0	3.51
	*Turdus rufiventris*	0	1	4.86	*Labidus praedator*	0.8	0	3.51
	*Tyrannus melancholicus*	0	1	4.86	*Solenopsis interrupta*	0.2	0.8	3.37
	*Volantinia jacarina*	1	0	4.86	*Mycocepurus goeldii*	0.8	0.2	3.08
	*Megarhynchus pitangua*	0	0.8	4.06				
	*Crypturellus parvirostris*	0.8	0	3.83				
	*Rhynchotus rufescens*	0.8	0	3.83				
	*Furnarius rufus*	0	0.8	3.62				
Eucalypt Forest(A) vs. Eucalypt Grassland(B)	*Guira guira*	1	0	5.76	*Pogonomyrmex coartactus*	0.8	0	4.14
	*Pitangus sulphuratus*	1	0	5.76	*Atta sexdens*	0.2	0.8	3.44
	*Turdus rufiventris*	1	0	5.76	*Wasmannia auropunctata*	0.8	0.2	3.24
	*Megarhynchus pitangua*	0.8	0	4.85				
	*Tyrannus melancholicus*	1	0.2	4.53				
	*Columbina talpacoti*	0.8	0	4.21				
Eucalypt Grassland(A) vs. Soybean Grassland(B)	*Ammodramus humeralis*	0	1	7.81	*Solenopsis interrupta*	1	0.2	3.92
	*Patagioenas picazuro*	1	0	7.81	*Paratrechina silvestrii*	0.2	1	3.75
	*Rhynchotus rufescens*	0	1	7.81	*Pachycondyla striata*	0	0.8	3.6
	*Tyrannus savana*	0	1	7.81	*Atta sexdens*	0.8	0	3.59
	*Zonotrichia capensis*	0.2	1	6.63	*Strumigenys lousianae*	0.2	1	3.48
	*Troglodytes aedon*	0.8	0	6.12	*Wasmannia auropunctata*	0.2	1	3.48
					*Ectatomma edentatum*	0.8	0.2	3

Species are ranked in order of importance (% contribution to the overall taxonomic distance between treatments) as determined by the SIMPER procedure. Only paired proportions ≥0.8 and ≤0.2 are reported.

Mantel tests showed that TBD, FBD and PBD were positively correlated for both bird and ant assemblages (Table 4). Partial correlations were also significant, except between bird functional and phylogenetic distances and between ant taxonomic and functional distances (Table 4). Then, taxonomic and phylogenetic distances were associated between ant and bird assemblages, independently of the distance between functional traits. For birds (but not for ants) taxonomic and functional distances were associated independently of the phylogenetic distance. For ants (but not for birds) functional and phylogenetic distances were associated independently of the taxonomic distance.

**Table 4:** Mantel correlation and partial correlation tests (p-values) between taxonomic, functional and phylogenetic distance matrices for bird and ant assemblages.

Test	Beta divers.	Taxonomic		Functional	
		Birds	Ants	Birds	Ants
Mantel correlation	Functional	0.98 (0.001)	0.69 (0.001)	---	---
	Phylogenetic	0.91 (0.001)	0.86 (0.001)	0.89 (0.001)	0.77 (0.001)
Partial Man. correlation	Functional	0.88 (0.001)	0.08 (0.149)	---	---
	Phylogenetic	0.40 (0.001)	0.70 (0.001)	0.04 (0.246)	0.49 (0.001)

Among bird traits, carnivorous, frugivorous-granivorous and insectivorous diet types, migratory-status, body-size, and high and low foraging-strata were conserved along the phylogeny (Table 5). Ant traits were all conserved along the phylogeny (Table 5).

**Table 5:** Phylogenetic conservatism tests for bird and ant functional traits.

	Trait	D_obs_	p(D_obs_<1)	p(D_obs_>0)
Birds	**CAR**	-1.01	0	0.9
	**FGR**	-0.39	0	0.776
	**MIG**	-0.08	0.015	0.563
	**SIZ**	0.03	0.007	0.493
	**HIGH**	0.03	0.001	0.503
	**LOW**	0.19	0.003	0.365
	**INS**	0.37	0.013	0.227
	OMN	0.58	0.132	0.156
	SHD	0.72	0.129	0.065
	TNA	0.97	0.441	0.009
	FOR	1.04	0.531	0.008
	GRA	1.13	0.662	0.005
	RP	1.21	0.789	0
	AHS	1.40	0.868	0.002
Ants	**W**	-4.76	0	0.981
	**SP**	-2.64	0	0.989
	**DD**	-2.57	0	0.985
	**SC**	-2.04	0	0.999
	**VF**	-1.56	0	0.999
	**GM**	-1.41	0	1
	**C**	-1.34	0	0.932
	**O**	-1.05	0	0.966
	**FG**	-0.94	0	0.999
	**T**	-0.82	0	0.999
	**S**	-0.63	0	0.988
	**SP**	-0.35	0	0.841
	**GRA**	-0.27	0.002	0.694
	**L**	-0.17	0	0.74
	**GP**	-0.07	0	0.585
	**FOR**	0.27	0	0.169
	**M**	0.40	0.003	0.2
	**GRA**	0.73	0.02	0.002

Traits are ranked by increasing observed D-statistic value (Dobs); p(D_obs_<1) is the significance level in the test of random distribution of traits along phylogeny, and p(D_obs_>0) in the test against the expected by the Brownian evolutionary model. For abbreviations see Table 1. Traits highly (D_obs_< 0) and moderately (0 < D_obs_< 1) conserved are bolded.

## Discussion

Studying simultaneously major regional (i.e. biome determined by climate) and local (i.e. habitat type determined by human land use) factors driving species distribution contributes to a unified view of community dynamics [93]. As expected, our results showed that bird and ant assemblages from both the Atlantic Forest and Pampean Grassland differed taxonomically, functionally and phylogenetically in response to local environmental conditions imposed by human land use (i.e., eucalypt plantations and soybean cropfields). Studies conducted in northern United State along local and regional environmental gradients also showed that composition of bird communities resulted from the interactive effects between land use and climate/geomorphology [94]. Consequently, changes in taxonomic, functional and phylogenetic compositions due to human land use should be interpreted accounting for the biome, at least in the Pampean Grassland and the Atlantic Forest and presumably in other ecoregions as well. It has been shown that species from the original regional pool respond to land use depending on the environmental similarity between the native and novel habitat [39, 95].

Results obtained from PERMANOVA, PERMDISP and the NMDS exploration indicated that the biome on which human activities developed had a significant influence on the capability of species to exploit different types of human-created habitats. Our studied biomes have different species pools over which soybean cropfields and eucalypt plantations imposed the additional environmental filter, which likely leaded to the differential taxonomic, functional and phylogenetic assemblages. Furthermore, human-created habitats may be more or less similar (or preserve more or less elements) to the biome in which the habitat is located. For example, eucalypt plantations were structurally more similar to forests than to grasslands; and soybean cropfields were more similar to grasslands than to forests. Thus, the set of traits phylogenetically distributed allows species to use soybean cropfields and eucalypt plantations differently depending on whether the human activity is developed in the Pampas Grassland or the Atlantic Forest. In a previous study we demonstrated that soybean cropfields supported a higher proportion of native bird species in Grassland than in Forest, where similarity in vegetation structure between the native and human-created habitat was greater; the opposite occurred in tree plantations that supported more native species in Forest than in Grasslands [39].

Results from PERMANOVA and PERMDISP indicated that both biome and land use were associated with taxonomic, functional and phylogenetic turnovers in bird and ant assemblages. Thus, land uses and biomes seemed to promote assemblage differentiation not only in species identities but in traits and lineages that occurred in anthropogenic habitats. Moreover, we showed evidence that TBD, FBD and PBD were positively associated to each other, and that most of the studied traits were conserved along bird and ant phylogenies. Results obtained by the partial Mantel tests indicated that in bird assemblages, species and trait compositions were not completely phylogenetically structured, and that other traits (not considered here) caused species and phylogenetic composition associations. However, the association between taxonomic and functional distances in ant communities was completely explained by the phylogenetic information considered. Moreover, the association between functional and phylogenetic distance in bird communities was completely explained by the taxonomic distance. Overall, those results suggest that taxonomic, functional and phylogenetic distances between assemblages were related to changes in phylogenetically conserved traits along each regional species pool. That is, lineages bearing traits which favored colonization and survival in soybean cropfields or eucalypt plantations differed between biomes, which is the pattern expected when communities were assembled by environmental filtering of independent lineages [1,21].

Bird TBD was associated (to some extent) with FBD beyond any phylogenetic structure of the communities. On the one hand, as mentioned before, that result is extremely dependent on the selected traits and consistent with traits that were not phylogenetically conserved. In fact, the inclusion of additional or alternative relevant traits could even reverse our findings if there is a phylogenetic signal in them. The result is also dependent on the obtained phylogenetic tree, because different phylogenetic considerations may lead to different trees and derive in a different result. On the other hand, it is possible that a “specific (likely ecological) component” (e.g., quantity and quality of available resources) explained species turnovers beyond any phylogenetic relationship. As expected, both birds and ants were sensitive to human alterations of the habitat such as changing vegetation structure [51,96–99]. Furthermore, the general patterns of turnover that we found were relatively similar between taxa regardless of differences between bird and ant life histories. Although previous studies showed that the combined effects of local and regional factors determined taxonomic, functional or phylogenetic compositions in assemblages of varied taxa [100–103], this is the first field study combining the analysis of the three beta diversity facets on both a vertebrate and invertebrate taxa.

Finally, we used an integrated approach to study community differentiation considering three complementary facets (taxonomic, functional and phylogenetic) of beta diversity. We accounted for regional (biome) and local (land use) factors that proved to influence turnover. To emphasize the relevance of the approach, we used conservation priority biomes and extended human-created habitats as the regional and local factors, respectively, influencing assemblage composition. In countries where economy depends on human activities that require large areas (e.g. agriculture, livestock, forestry), our results contribute to the development of sound land use planning and beta diversity conservation. For example, our results should be useful to help selecting the most appropriate ecoregion to develop agriculture and forestry in Argentina. We hope our work will serve to encourage the use of a more complete approach to the study of beta diversity and its application in conservation biology.

## Supporting Information

S1 Fig Functional dendrograms and phylogenetic trees for the recorded bird and ant species in the Atlantic forest and the Pampas grassland. (DOC)

S1 Table Coordinates of birds and ants sampling sites in the Atlantic Forest and the Pampas grassland of Argentina and Paraguay. (DOC)

S2 Table Taxonomic keys used to identify ant species or morphospecies in the Atlantic forest and the Pampas grassland of Argentina and Paraguay. (DOC)

S3 Table Ant and bird species recorded in both regions and land uses. (DOC)
