# Supplementary material for: Integrating Taxonomic, Functional and Phylogenetic Beta Diversities: Interactive Effects with the Biome and Land Use across Taxa
Source: PLoS One. 2015 May 15;10(5):e0126854. doi: 10.1371/journal.pone.0126854 (PMC4433125; doi:10.1371/journal.pone.0126854)
Supplement: S1 Fig — (DOC) [file pone.0126854.s001.doc]

**S1 Fig**. Functional dendrograms and phylogenetic trees for the recorded bird and ant species in the Atlantic forest and Pampean grassland

Functional dendrogram for the recorded bird species


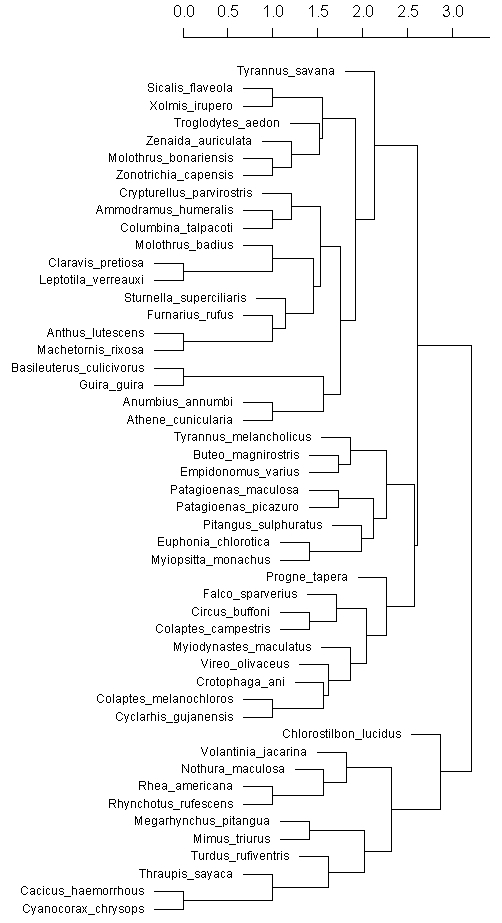


Functional dendrogram for the captured ant species/morphospecies.


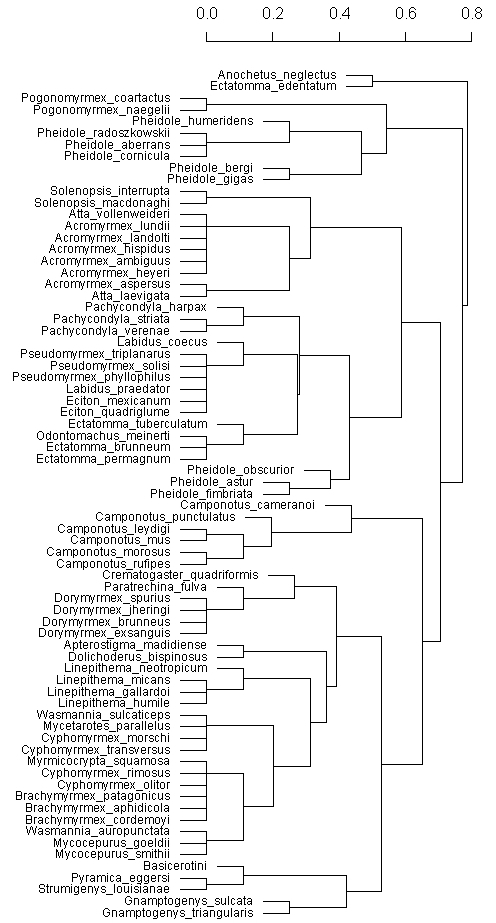


Phylogenetic tree assembled with the recorded bird species

Phylogenetic tree assembled with the captured ant species/morphospecies.
