# Supplementary material for: Integrating Taxonomic, Functional and Phylogenetic Beta Diversities: Interactive Effects with the Biome and Land Use across Taxa
Source: PLoS One. 2015 May 15;10(5):e0126854. doi: 10.1371/journal.pone.0126854 (PMC4433125; doi:10.1371/journal.pone.0126854)
Supplement: S1 Table — (DOC) [file pone.0126854.s002.doc]

**S1 Table:** Coordinates of birds and ants sampling sites in soybean cropfields and eucalypt plantations in the Atlantic Forest and the Pampas grassland of Paraguay and Argentina.

| **Taxa** | **Region** | **Land use** | **Latitude (Easting)** | **Longitude (Northing)** |
| --- | --- | --- | --- | --- |
| Birds | Grassland | Eucalypt | 373181 | 6473967 |
| Birds | Grassland | Eucalypt | 398694 | 6499607 |
| Birds | Grassland | Eucalypt | 400045 | 6499890 |
| Birds | Grassland | Eucalypt | 388571 | 6510623 |
| Birds | Grassland | Eucalypt | 380515 | 6461235 |
| Birds | Forest | Eucalypt | 735022 | 7201151 |
| Birds | Forest | Eucalypt | 735764 | 7203357 |
| Birds | Forest | Eucalypt | 725344 | 7195346 |
| Birds | Forest | Eucalypt | 714934 | 7194853 |
| Birds | Forest | Eucalypt | 722924 | 7194725 |
| Birds | Grassland | Soybean | 380127 | 6468018 |
| Birds | Grassland | Soybean | 378580 | 6466206 |
| Birds | Grassland | Soybean | 378675 | 6464296 |
| Birds | Grassland | Soybean | 377442 | 6464309 |
| Birds | Grassland | Soybean | 376432 | 6467958 |
| Birds | Forest | Soybean | 720616 | 7192867 |
| Birds | Forest | Soybean | 721912 | 7192480 |
| Birds | Forest | Soybean | 716425 | 7200130 |
| Birds | Forest | Soybean | 717976 | 7199907 |
| Birds | Forest | Soybean | 721365 | 7190981 |
| Ants | Grassland | Eucalypt | 398866 | 6499738 |
| Ants | Grassland | Eucalypt | 399993 | 6499907 |
| Ants | Grassland | Eucalypt | 371172 | 6472803 |
| Ants | Grassland | Eucalypt | 369993 | 6474134 |
| Ants | Grassland | Eucalypt | 373152 | 6473862 |
| Ants | Forest | Eucalypt | 723510 | 7195031 |
| Ants | Forest | Eucalypt | 724985 | 7195926 |
| Ants | Forest | Eucalypt | 715422 | 7194852 |
| Ants | Forest | Eucalypt | 735564 | 7202812 |
| Ants | Forest | Eucalypt | 734617 | 7201066 |
| Ants | Grassland | Soybean | 379737 | 6467568 |
| Ants | Grassland | Soybean | 378587 | 6466572 |
| Ants | Grassland | Soybean | 377385 | 6466489 |
| Ants | Grassland | Soybean | 378406 | 6463817 |
| Ants | Grassland | Soybean | 377437 | 6463770 |
| Ants | Forest | Soybean | 717316 | 7200333 |
| Ants | Forest | Soybean | 718330 | 7199551 |
| Ants | Forest | Soybean | 720454 | 7193228 |
| Ants | Forest | Soybean | 721532 | 7191074 |
| Ants | Forest | Soybean | 721838 | 7192047 |
