# Supplementary material for: Integrating Taxonomic, Functional and Phylogenetic Beta Diversities: Interactive Effects with the Biome and Land Use across Taxa
Source: PLoS One. 2015 May 15;10(5):e0126854. doi: 10.1371/journal.pone.0126854 (PMC4433125; doi:10.1371/journal.pone.0126854)
Supplement: S2 Table — (DOC) [file pone.0126854.s003.doc]

**S2 Table.** Taxonomic keys used to identify ant species or morphospecies in the Atlantic Forest and the Pampas grassland of Argentina and Paraguay.

Fowler, H.G. (1988). Taxa of the Neotropical grass-cutting ants, *Acromyrmex* (Moellerius) (Hymenoptera: Formicidae: Attini). Cientifica, Sao Paulo 16, 281-296.

Kempf, W.W. (1963). A review of the ant genus *Mycocepurus* Forel, 1893 (Hymenoptera: Formicidae). Studia Entomologica, 6, 417-432.

Kempf, W.W. (1964). A revision of the Neotropical fungus-growing ants of the genus *Cyphomyrmex* Mayr. Part I. Group of strigatus Mayr (Hym., Formicidae). Studia Entomologica, 7, 1-44.

Kempf, W.W. (1966). A revision of the Neotropical fungus-growing ants of the genus *Cyphomyrmex* Mayr. Part II. Group of rimosus (Spinola) (Hym. Formicidae). Studia Entomologica, 8, 161-200.

Kusnezov, N. (1949). El género *Cyphomyrmex* (Hymenoptera, Formicidae) en la Argentina. Acta Zoologica Lilloana, 8, 427-456.

Kusnezov, N. (1951). El género *Pogonomyrmex* Mayr (Hym., Formicidae). Acta Zoologica Lilloana, 11, 227-333.

Kusnezov, N. (1952). El género *Camponotus* en la Argentina (Hymenoptera, Formicidae). Acta Zoologica Lilloana, 12, 183-252.

Kusnezov, N. (1952). El género *Wasmannia* en la Argentina (Hymenoptera, Formicidae). Acta Zoologica Lilloana, 10, 173-182.

Kusnezov, N. (1952) El género *Pheidole* en la Argentina (Hymenoptera, Formicidae). Acta Zoologica Lilloana, 12, 2-88.

Kunezov, N. (1978). Hormigas Argentinas: Clave para su identificación. Edición preparada por R. Golbach, Fundación Miguel Lillo, Miscelánea, 61.

González-Campero, M.C. & Elizalde, L. (2008). A new species of *Anochetus* (Hymenoptera: Formicidae: Ponerini) from Argentina and Paraguay, associated with a leaf cutter ants. Entomotropica, 23, 97-102.

Gonçalves, C.R. (1942). Contribuição para o conhecimento do gênero *Atta* Fabr., das formigas saúvas. Boletim da Sociedade Brasileira de Agronomia, 5, 333-358.

Gonçalves, C.R. (1961). O genero *Acromyrmex* no Brasil (Hym. Formicidae). Studia Entomologica, 4, 113-180.

Jiménez, E., Fernández, F., Arias, T.M. & Lozano-Zambrano, F. H. (2007). Sistemática, biogeografía y conservación de las hormigas cazadoras de Colombia. Instituto de Investigación de Recursos Biológicos Alexander von Humboldt. Bogotá D. C., Colombia

Lattke, J. E. (1997). Revisión del Género *Apterostigma* Mayr (Hymenoptera: Formicidae). Arquivos Zoología Sao Paulo, 34, 121-221.

Lattke, J. & Goitia, W. (1997). El género *Strumigenys* (Hymenoptera: Formicidae) en Venezuela. Caldasia, 19, 367-396.

Mayhé-Nunes, A.J. (1995). Sinopse do genero *Mycetarotes* (Hym., Formicidae), com a descriçao de duas especies novas. Bol. Entomol. Venez., 10, 197-205.

Mayhé-Nunes, A.J. & Brandão, C.R.F. (2002). Revisionary studies on the attine ant genus *Trachymyrmex* Forel. Part 1: Definition of the genus and the opulentus group (Hymenoptera: Formicidae). Sociobiology, 40, 667-698.

Mayhé-Nunes, A.J. & Brandão, C.R.F (2005). Revisionary studies on the attine ant genus *Trachymyrmex* Forel. Part 2: The Iheringi group (Hymenoptera: Formicidae). Sociobiology, 45, 271-305.

Mayhé-Nunes, A.J. & Brandão, C.R.F (2007). Revisionary studies on the attine ant genus *Trachymyrmex* Forel. Part 3: The Jamaicensis group (Hymenoptera: Formicidae). Zootaxa, 1444, 1-21.

Taber, S.W. (1998). The world of the harvester ants. Texas A & M University Press, College Station.

The Ants of North America -William and Emma Mackay Centennial Museum, Laboratory for Environmental Biology, The University of Texas, El Paso, TX http://www.utep.edu/leb/antgenera.htm

Trager, J.C. (1991). A revision of the fire ants, *Solenopsis geminata* group (Hymenoptera: Formicidae, Myrmicinae). Journal of the New York Entomological Society, 99, 141-198.

Quirán, E. M., Martínez, J.J. & Bachmann, A.O. (2004). El género *Brachymyrmex* Mayr en la Argentina. I. Acta Zool. Mex., 20, 273-285.

Quirán, E. M. (2005). El género Neotropical *Brachymyrmex* Mayr, 1868 (Hymenoptera: Formicidae) en la Argentina. II. Redescripción de las especies, *B. admotus* Mayr; *B. brevicornis* Emery y *B. gaucho* Santschi. Neotrop. Entomol., 34, 761-768.

Quirán, E.M. (2007). El género *Brachymyrmex* Mayr en la Argentina. III. Redescripción de *B. aphidicola* Forel, *B. australis* Forel y *B. constrictus* Santschi. Neotrop. Entomol., 36, 699-706.

Wild, A.L. (2002). The genus *Pachycondyla* (Hymenoptera: Formicidae) in Paraguay. Boletín del Museo Nacional de Historia Natural del Paraguay, 14, 1-18.

Wild, A.L. (2007). Taxonomic revision of the ant genus *Linepithema* (Hymenoptera: Formicidae). University of California Publications in Entomology, 126, 1-159.

Wilson, E.O. (2003). *Pheidole* in the New World. A dominant, hyperdiverse ant genus. Harvard University Press, Cambridge, MA.
