# Supplementary material for: Integrating Taxonomic, Functional and Phylogenetic Beta Diversities: Interactive Effects with the Biome and Land Use across Taxa
Source: PLoS One. 2015 May 15;10(5):e0126854. doi: 10.1371/journal.pone.0126854 (PMC4433125; doi:10.1371/journal.pone.0126854)
Supplement: S3 Table — (DOC) [file pone.0126854.s004.doc]

**S3 Table.** Ant and bird recorded species in both biomes and land uses

| **Species** | | **Grassland** | | | | **Forest** | | |
| --- | --- | --- | --- | --- | --- | --- | --- | --- |
|  |  | **Eucalypt** | | **Soybean** | | **Eucalypt** | | **Soybean** |
| **Ants** |  | |  | |  | |  | |
| *Acanthostichus* |  | | X | |  | |  | |
| *Acromyrmex ambiguus* | X | |  | |  | |  | |
| *Acromyrmex aspersus* | X | |  | | X | |  | |
| *Acromyrmex heyeri* |  | | X | |  | |  | |
| *Acromyrmex hispidus* | X | |  | | X | | X | |
| *Acromyrmex landolti* | X | |  | | X | |  | |
| *Acromyrmex lundi* | X | | X | |  | |  | |
| *Anochetus neglectus* |  | |  | | X | | X | |
| *Apterostigma madidiense* |  | |  | | X | | X | |
| *Atta capiguara-goiania* |  | |  | |  | | X | |
| *Atta laevigata* |  | |  | |  | | X | |
| *Atta sexdens* |  | | X | | X | | X | |
| *Atta vollenweideri* |  | |  | |  | | X | |
| *Basicerotini* |  | |  | | X | |  | |
| *Brachymyrmex aphidicola* | X | |  | |  | |  | |
| *Brachymyrmex brevicornis* | X | | X | | X | | X | |
| *Brachymyrmex cordemoyi* | X | | X | | X | | X | |
| *Brachymyrmex fiebrigi* | X | | X | | X | |  | |
| *Brachymyrmex nigricans* |  | |  | | X | |  | |
| *Brachymyrmex patagonicus* | X | |  | | X | | X | |
| *Brachymyrmex pictus* |  | |  | | X | |  | |
| *Camponotus cameranoi* | X | | X | |  | |  | |
| *Camponotus leydigi* | X | | X | | X | |  | |
| *Camponotus morosus* |  | | X | |  | |  | |
| *Camponotus mus* |  | | X | |  | |  | |
| *Camponotus punctulatus* | X | |  | |  | |  | |
| *Camponotus rufipes* | X | | X | | X | |  | |
| *Camponotus rusticus* |  | |  | | X | |  | |
| *Camponotus wheeleri* |  | |  | | X | |  | |
| *Cephalotes clypeatus* | X | |  | |  | |  | |
| *Crematogaster quadriformis* | X | | X | | X | |  | |
| *Crematogaster scelerata* | X | | X | |  | |  | |
| *Cyphomyrmex bruchi* |  | | X | |  | |  | |
| *Cyphomyrmex lilloanus* |  | | X | |  | |  | |
| *Cyphomyrmex morschi* | X | |  | |  | |  | |
| *Cyphomyrmex olitor* | X | |  | |  | | X | |
| *Cyphomyrmex rimosus* | X | | X | | X | |  | |
| *Cyphomyrmex transversus* | X | |  | | X | |  | |
| *Dolichoderus bispinosus* |  | |  | |  | | X | |
| *Dorymyrmex brunneus* |  | |  | | X | | X | |
| *Dorymyrmex exsanguis* | X | |  | |  | | X | |
| *Dorymyrmex jheringi* |  | | X | |  | | X | |
| *Dorymyrmex spurius* |  | |  | |  | | X | |
| *Eciton mexicanum* |  | |  | | X | | X | |
| *Eciton quadriglume* |  | |  | | X | | X | |
| *Ectatomma bruneum* |  | |  | | X | | X | |
| *Ectatomma edentatum* | X | | X | | X | | X | |
| *Ectatomma permagnum* |  | |  | | X | |  | |
| *Ectatomma tuberculatum* |  | |  | | X | |  | |
| *Gnamptogenys sulcata* | X | |  | | X | |  | |
| *Gnamptogenys triangularis* | X | |  | |  | |  | |
| *Labidus coecus* |  | |  | | X | |  | |
| *Labidus praedator* |  | |  | | X | | X | |
| *Linepithema gallardoi* | X | |  | |  | |  | |
| *Linepithema humile* | X | | X | | X | | X | |
| *Linepithema micans* | X | | X | | X | | X | |
| *Linepithema neotropicum* |  | |  | | X | |  | |
| *Mycetarotes parallelus* |  | |  | | X | | X | |
| *Mycocepurus goeldii* |  | | X | | X | | X | |
| *Mycocepurus smithi* |  | |  | |  | | X | |
| *Myrmicocrypta squamosa(cf)* | X | |  | |  | | X | |
| *Neivamyrmex pertyi* | X | | X | |  | |  | |
| *Nomamyrmex hartigi* |  | |  | | X | | X | |
| *Odontomachus meinerti* |  | |  | | X | |  | |
| *Pachycondyla harpax* |  | |  | | X | |  | |
| *Pachycondyla striata* | X | |  | | X | |  | |
| *Pachycondyla verenae* |  | |  | | X | |  | |
| *Paratrechina fulva* | X | |  | |  | |  | |
| *Paratrechina silvestrii* | X | |  | | X | | X | |
| *Pheidole aberrans* | X | | X | |  | |  | |
| *Pheidole astur* |  | |  | | X | |  | |
| *Pheidole bergi* | X | |  | | X | | X | |
| *Pheidole cornicula* |  | |  | |  | | X | |
| *Pheidole fimbriata* |  | |  | |  | | X | |
| *Pheidole gigas* | X | |  | |  | |  | |
| *Pheidole humeridens* | X | | X | |  | |  | |
| *Pheidole nubila* | X | | X | |  | | X | |
| *Pheidole obscurior* | X | |  | |  | |  | |
| *Pheidole pampana* | X | | X | | X | |  | |
| *Pheidole radozowsky* | X | | X | | X | | X | |
| *Pheidole rudigenis* | X | |  | |  | |  | |
| *Pheidole trageri* |  | |  | |  | | X | |
| *Pogonomyrmex coartactus* |  | | X | |  | |  | |
| *Pogonomyrmex naegelli* | X | | X | | X | | X | |
| *Pseudomyrmex phyllophilus* | X | |  | |  | |  | |
| *Pseudomyrmex solisi* | X | |  | |  | |  | |
| *Pseudomyrmex triplanarus* | X | |  | |  | |  | |
| *Pyramica eggersi* |  | |  | | X | | X | |
| *Sericomyrmex* |  | |  | |  | | X | |
| *Solenopsis electra* |  | | X | |  | |  | |
| *Solenopsis interrupta* | X | | X | | X | | X | |
| *Solenopsis macdonaghi* |  | | X | | X | |  | |
| *Solenopsis quinquecuspis* |  | | X | |  | |  | |
| *Strumigenys lousianae* | X | |  | |  | | X | |
| *Strumigenys oglobini* |  | | X | |  | | X | |
| *Trachymyrmex cirratus* | X | | X | |  | | X | |
| *Trachymyrmex sp1* | X | | X | | X | |  | |
| *Wasmannia auropunctata* | X | | X | | X | | X | |
| *Wasmannia sulcaticeps* | X | | X | |  | |  | |
| **Birds** |  | |  | |  | |  | |
| *Ammodramus humeralis* |  | | X | |  | | X | |
| *Anthus lutescens* |  | |  | |  | | X | |
| *Anumbius annumbi* |  | | X | |  | |  | |
| *Athene cunicularia* |  | |  | |  | | X | |
| *Basileuterus culicivorus* |  | |  | | X | |  | |
| *Buteo magnirostris* | X | |  | | X | |  | |
| *Cacicus haemorrhous* |  | |  | | X | |  | |
| *Chlorostilbon lucidus* |  | |  | | X | |  | |
| *Circus buffoni* |  | |  | |  | | X | |
| *Claravis pretiosa* |  | |  | | X | |  | |
| *Colaptes campestris* |  | | X | | X | |  | |
| *Colaptes melanochloros* |  | |  | | X | |  | |
| *Patagioenas maculosa* |  | |  | | X | |  | |
| *Patagioenas picazuro* | X | |  | | X | |  | |
| *Columbina talpacoti* |  | |  | | X | | X | |
| *Crotophaga ani* |  | |  | | X | |  | |
| *Crypturellus parvirostris* |  | |  | |  | | X | |
| *Cyanocorax chrysops* |  | |  | | X | |  | |
| *Cyclarhis gujanensis* |  | |  | | X | |  | |
| *Empidonomus varius* |  | |  | | X | |  | |
| *Euphonia chlorotica* |  | |  | | X | |  | |
| *Falco sparverius* |  | | X | |  | | X | |
| *Furnarius rufus* | X | | X | | X | |  | |
| *Guira guira* |  | |  | | X | |  | |
| *Leptotila verreauxi* | X | |  | |  | |  | |
| *Machetornis rixosa* |  | |  | | X | |  | |
| *Megarhynchus pitangua* |  | |  | | X | |  | |
| *Mimus triurus* |  | | X | |  | |  | |
| *Molothrus badius* |  | | X | |  | |  | |
| *Molothrus bonariensis* |  | | X | |  | |  | |
| *Myiodynastes maculatus* |  | |  | | X | |  | |
| *Myiopsitta monachus* | X | |  | |  | |  | |
| *Nothura maculosa* |  | | X | |  | |  | |
| *Pitangus sulphuratus* |  | | X | | X | |  | |
| *Progne tapera* |  | | X | |  | |  | |
| *Rhea americana* |  | | X | |  | |  | |
| *Rhynchotus rufescens* |  | | X | |  | | X | |
| *Sicalis flaveola* |  | |  | | X | |  | |
| *Sturnella superciliaris* |  | | X | |  | | X | |
| *Thraupis sayaca* | X | |  | | X | | X | |
| *Tringa solitaria* |  | | X | |  | |  | |
| *Troglodytes aedon* | X | |  | | X | |  | |
| *Turdus rufiventris* |  | |  | | X | |  | |
| *Tyrannus melancholicus* | X | | X | | X | |  | |
| *Tyrannus savana* |  | | X | | X | | X | |
| *Vireo olivaceus* | X | |  | | X | |  | |
| *Volantinia jacarina* |  | |  | |  | | X | |
| *Xolmis irupero* |  | | X | |  | |  | |
| *Zenaida auriculata* |  | | X | |  | | X | |
| *Zonotrichia capensis* | X | | X | |  | |  | |
| *Ammodramus humeralis* |  | | X | |  | | X | |
| *Anthus lutescens* |  | |  | |  | | X | |
